# Two Dynamin-2 Genes Are Required for Normal Zebrafish Development

**DOI:** 10.1371/journal.pone.0055888

**Published:** 2013-02-13

**Authors:** Elizabeth M. Gibbs, Ann E. Davidson, Arden Trickey-Glassman, Carey Backus, Yu Hong, Stacey A. Sakowski, James J. Dowling, Eva L. Feldman

**Affiliations:** 1 Department of Neurology, University of Michigan, Ann Arbor, Michigan, United States of America; 2 Neuroscience Program, University of Michigan, Ann Arbor, Michigan, United States of America; 3 Department of Pediatrics & Communicable Diseases, University of Michigan, Ann Arbor, Michigan, United States of America; 4 A. Alfred Taubman Medical Research Institute, University of Michigan, Ann Arbor, Michigan, United States of America; Instituto de Ciencia de Materiales de Madrid - Instituto de Biomedicina de Valencia, Spain

## Abstract

Dynamin-2 (DNM2) is a large GTPase involved in clathrin-mediated endocytosis and related trafficking pathways. Mutations in human *DNM2* cause two distinct neuromuscular disorders: centronuclear myopathy and Charcot-Marie-Tooth disease. Zebrafish have been shown to be an excellent animal model for many neurologic disorders, and this system has the potential to inform our understanding of DNM2-related disease. Currently, little is known about the endogenous zebrafish orthologs to human DNM2. In this study, we characterize two zebrafish dynamin-2 genes, *dnm2* and *dnm2-like*. Both orthologs are structurally similar to human DNM2 at the gene and protein levels. They are expressed throughout early development and in all adult tissues examined. Knockdown of *dnm2* and *dnm2-like* gene products resulted in extensive morphological abnormalities during development, and expression of human *DNM2* RNA rescued these phenotypes. Our findings suggest that *dnm2* and *dnm2-like* are orthologs to human DNM2, and that they are required for normal zebrafish development.

## Introduction

Dynamins are large GTPases involved in a wide range of cell and organelle fission events. The dynamin superfamily is made up of classical dynamins and dynamin-like proteins. Classical dynamins are critical components of clathrin-mediated endocytosis, where they contribute to the release of newly formed endosomes [1], [2], [3]. In addition to this well-characterized role in endocytosis, classical dynamins also participate in a variety of membrane trafficking functions including phagocytosis, caveolae internalization, and trans-Golgi transport [4], [5], [6]. In mammals, there are three classical dynamins: dynamin-1 (DNM1), dynamin-2 (DNM2), and dynamin-3 (DNM3). Of these three genetic isoforms, only DNM2 is ubiquitously expressed [7], [8], [9] and a requirement for DNM2 during development is evidenced by an embryonic lethal phenotype in *Dnm2* knockout mice [10]. Furthermore, mutations in human *DNM2* also cause two different neuromuscular disorders; Charcot-Marie-Tooth disease and centronuclear myopathy [11], [12].

Currently, there is no published characterization of any classical dynamin in the zebrafish genome. Given the prominent role of DNM2 in cellular function and human disease, characterizing the endogenous zebrafish dynamin-2 is an important task. Several studies of zebrafish endocytosis have utilized putative markers or inhibitors of dynamin-2; however, none of these reports examined functional or structural similarity between human DNM2 and a zebrafish homolog [13], [14], [15]. Establishing this orthologous relationship will enable future studies of endocytosis and other dynamin-related pathways in the zebrafish.

In this study, we characterize two zebrafish dynamin-2 genes, *dnm2* and *dnm2-like*. We demonstrate that *dnm2* and *dnm2-like* are structurally similar to human *DNM2* at both the gene and protein levels, and that these gene products are ubiquitously expressed in adult tissue. Using morpholino-mediated knockdown, we show that depletion of *dnm2* and *dnm2-like* gene products causes morphological abnormalities during development. We further show that knockdown of *dnm2* results in substantial motor defects and histological abnormalities in larval muscle. Overexpression of human *DNM2* mRNA is able to rescue both *dnm2* and *dnm2-like* phenotypes. Taken together, this evidence suggests that *dnm2 and dnm2-like* are structural and functional orthologs to human DNM2, and that they are required for normal embryonic development in the zebrafish.

## Materials and Methods

### Phylogenetic and Syntenic Analysis

Multiple species alignments and phylogenetic analyses were performed using Mega 5.1 software [16]. Phylogenies were created using the neighbor-joining method with 1000 bootstrap replicates. Syntenic genes were identified using NCBI and Ensembl databases, and orthology of these genes was confirmed using reciprocal BLAST searches against the human and zebrafish genomes.

### Animal Care and Ethics Statement

Zebrafish (AB strain) were bred and raised according to established protocols. Experiments were performed on zebrafish embryos and larvae between 1 and 2 days post fertilization (dpf). All animals were handled in strict accordance with good animal practice as defined by national and local animal welfare bodies, and all animal work was approved by the appropriate committee (University of Michigan UCUCA #09835).

### RACE-PCR and RT-PCR

Rapid amplification of cDNA end (RACE) was performed to confirm the 3′ sequence of zebrafish *dnm2* using the 3′-RACE GeneRacer kit (Invitrogen) according to the manufacturer’s protocol. To clone *dnm2*, total RNA was extracted from 2 dpf larvae using an RNeasy kit (Qiagen). For expression studies, RNA was extracted from adult zebrafish and embryos at various developmental timepoints. For analysis of morpholino-mediated knockdown, RNA was extracted from morpholino-injected and control larvae at 2 dpf. cDNA was synthesized from RNA using the iScript cDNA Synthesis kit (Bio-Rad). PCR was performed on a MyCycler thermocycler (BioRad) using GoTaq Green 2x Master Mix (Promega) and the following primers: 5′-TCACCCTGGGAGTGAAACAGC-3′ (*ef1α* forward), 5′-ACTTGCAGGCGATGTGAGCAG-3′ (*ef1α* reverse), 5′-GGCCAAAGTTGTAACCTGGA-3′ (*dnm2* forward), 5′-CGGTTTCTGCTTCAATCTCC-3′ (*dnm2* reverse), 5′-TTGTGGACTTTGACGAGGTTCGGA (*dnm2-like* forward), 5′-ATGCTGGATGGGACAGGAAGAACT-3′ (*dnm2-like* reverse), 5′-ACACGGAGCAGAGAAACGTCTACA-3′ (human *DNM2* forward), and 5′-GGTGCATGATGGTCTTTGGCATGA-3′ (human *DNM2* reverse).

### RNA Synthesis

Wild-type human DNM2 plasmid was purchased from Invitrogen (ORF Gateway® Entry IOH53617). Expression vectors were generated by recombination of DNM2 with p5E-CMV/SP6, p3E-polyA, and pDestTol2pA2 cassettes from the Tol2kit v1.2, a kind gift of Dr. Chi-Bin Chien [17]. Gateway recombination reactions were performed using LR Clonase II Plus Enzyme Mix (Invitrogen). The DNM2 rescue plasmid was linearized with NotI and transcribed using the SP6 mMessage Machine kit (Ambion).

### Morpholino and RNA Injection of Zebrafish Embryos

For *dnm2* and *dnm2-like* knockdown, the following custom splice-targeting morpholinos were designed and purchased, along with standard control morpholino, from Gene Tools: 5′-TGCCGTGCTCATTAACACACTCACC-′3 (*dnm2* MO), 5′-CAACCCCACTGCTCTCACCGGATCT-3′ (*dnm2-like* MO), and 5′-CCTCTTACCTCAGTTACAATTATA-3′ (GeneTools standard control). Fertilized eggs were collected after timed mating of adult zebrafish and injected at the 1–2-cell stage using a Nanoject II injector (Drummond Scientific). Embryos were injected with *dnm2-like* MO (0.1 mM) or *dnm2* MO (0.3 mM) in a 4.6 nL volume. Injection of control morpholino (ctl MO; 0.3 mM) verifies that the described injections at this concentration do not confer morpholino-mediated toxicity, and the same morpholino concentrations were utilized in all experiments. For rescue experiments, embryos were co-injected with human *DNM2* RNA (30 ng/µl). Larvae were photographed using a Nikon AZ-100 microscope or a Leica MXIII Stereoscope.

### Analysis of Motor Behavior

Spontaneous coiling was measured at 1 dpf by observing the number of coils in a 60 second period. Touch-evoked motor behaviors were measured in 3 dpf larvae by touching the tail with a pair of No. 5 forceps. Larvae that did not swim following three consecutive tail stimuli were recorded as “no response”.

### Histopathologic Analysis

For semi-thin sections, zebrafish were fixed overnight in Karnovsky’s fixative at 3 dpf and then processed for embedding in epon by the Microscopy and Imaging Laboratory core facility at the University of Michigan. Semi-thin sections were stained with toluidine blue and photographed using an Olympus BX43 microscope. Myofiber size was determined by measuring the length of two continuous myofibers spanning the first myosepta caudal to the yolk sac using Adobe Photoshop evaluation of photomicrographs from semi-thin sections. Electron microscopy was performed using a Phillips CM-100 transmission electron microscope as previously described [18].

### In situ Hybridization

*In situ* hybridization against *dnm2* was performed as described previously [18]. Probes were made by *in vitro* transcription with T7 or SP6 RNA polymerase (Promega), using templates generated by PCR. Probe template was generated by PCR using the following primers: 5′-ATTTAGGTGACACTATAGA CTGCTGCAGATGGTCCAGCAATTT-3′ (Forward, SP6), and 5′-TAATACGACTCACTATAGG TTTCTCAGGGTAAACGCCTGCTCT-3′ (Reverse, T7). PCR was performed on cDNA from 1 dpf wild-type (AB) embryos, and probe template sequence was verified by sequencing.

### Statistical Analysis

Statistical analysis was performed on data using the GraphPad Prism 5 software package. Significance was determined using ANOVA or Fisher’s exact test.

## Results

### Structure and Organization of two Dynamin-2 Genes in Zebrafish

**Figure 1:**
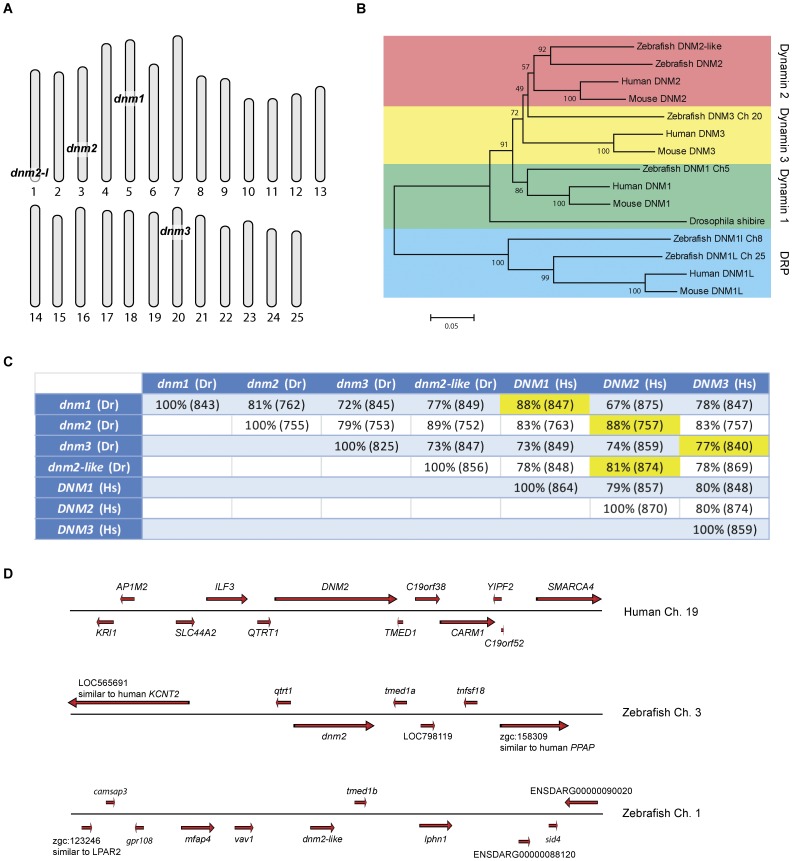
Phylogenetic and syntenic analysis of *dnm2* and *dnm2-like.* (A) Chromosomal locations of zebrafish homologues to human *DNM1*, *DNM2* and *DNM3*. (B) Phylogenetic tree comparing dynamin-2 genes in multiple species. (C) Comparison of zebrafish classical dynamins with human classical dynamins. Percent identity was determined by BLASTP. The length of homologous overlap is in parenthesis (number of amino acids). (D) Syntenic organization of human *DNM2* compared with zebrafish *dnm2* and *dnm2-like*.

Using public databases (NCBI, ENSEMBL, ZFIN) and RACE-PCR, we identified two separate zebrafish genes, *dnm2* and *dnm2-like*, which are highly related to human *DNM2,* on chromosomes 3 and 1 (Figure 1A; Genbank ID559334 and ID 406525; zfin zgc:114072 and zgc:77233). 3′ RACE-PCR on *dnm2* identified an additional 3 exons not included in any databases. These exons shared sequence homology with the 3 final exons in human *DNM2* and zebrafish *dnm2-like*. We additionally screened these databases for zebrafish genes with high sequence homology to other human classical dynamins. Comparison of the two putative zebrafish genes with human dynamins revealed that both *dnm2* and *dnm2-like* share highest sequence homology with human *DNM2* (Figure 1C). Phylogenetic analysis also grouped both genes into the *DNM2* cluster (Figure 1B). Analysis of genes surrounding the human *DNM2* revealed a conserved syntenic cluster including the *dnm2* gene on zebrafish chromosome 3 (Figure 1D).

**Figure 2:**
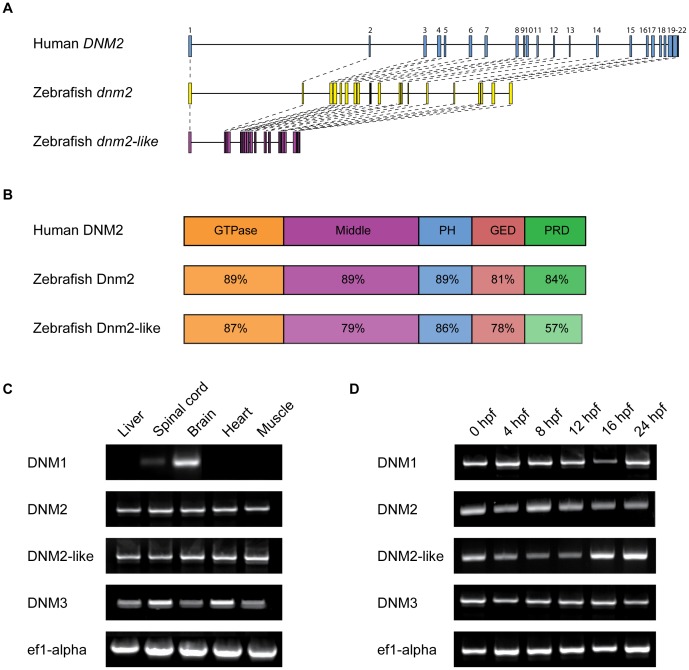
Structure and expression of *dnm2* and *dnm2-like.* (A) Molecular intron-exon organization of human *DNM2,* zebrafish *dnm2* and zebrafish *dnm2-like.* (B) Protein structure of zebrafish Dnm2 and Dnm2-like compared to human DNM2. Percent identity between zebrafish and human protein domains was calculated using BLASTP. PH, pleckstrin homology domain; GED, GTPase effector domain; PRD, proline-rich domain. (C) RT-PCR was used to assay spatial expression levels of *dnm2* and *dnm2-like* in tissues isolated from adult zebrafish. Primers for *ef1α* were used as an internal control. (D) RT-PCR was used to assay temporal expression levels of *dnm2* and *dnm2-like* between 0 hpf and 24 hpf. All classical dynamins appear to be deposited as maternal mRNAs and expressed throughout early development.

Both zebrafish proteins share all five major domains of human DNM2, including a GTPase domain, a GTPase effector domain (GED), a dynamin-specific middle domain, a pleckstrin homology (PH) domain, and a proline-rich domain (PRD). The two zebrafish *dnm2* genes share similar intron-exon organization with human *DNM2*, although *dnm2-like* has substantially smaller introns than either other gene (Figure 2A). At the protein level, these domains all share close identity with the domains of human DNM2 (Figure 2B).

### *dnm2* and *dnm2-like* Genes are Widely Expressed in Adult and Embryonic Tissue

To determine the expression pattern of *dnm2* and *dnm2-like*, we performed RT-PCR on adult zebrafish tissues and whole zebrafish larvae at several time points. Both *dnm2* and *dnm2-like* mRNA was detected in all adult tissues examined (Figure 2C). Both genes products were also detected at the earliest stages of development, indicating that both *dnm2* and *dnm2-like* are likely maternally deposited mRNAs (Figure 2D). Ubiquitous *dnm2* expression was additionally confirmed by *in situ* hybridization in 1 dpf embryos (Figure S1).

### Morpholino-mediated Knockdown of Zebrafish *dnm2* and *dnm2-like* Gene Expression

**Figure 3:**
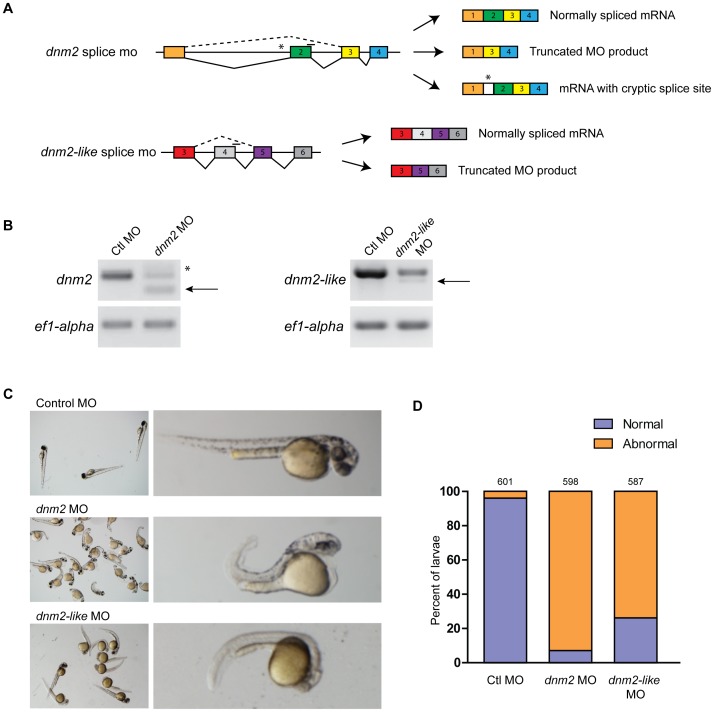
Morpholino-mediated knockdown of *dnm2* and *dnm2-like* expression results in morphological changes. (A) Splice targeting morpholinos were designed against intron-exon boundaries within the *dnm2* and *dnm2-like* genes. (B) Knockdown in morpholino injected embryos was verified using RT-PCR. Embryos were injected with a scrambled control morpholino (Ctl MO; 0.3 mM), *dnm2* MO (0.3 mM), or *dnm2-like* MO (0.1 mM). Arrows indicate the alternative splice product induced by *dnm2* MO and *dnm2-like* MO injection. *dnm2* MO injection also resulted in an additional higher weight band due to activation of a cryptic splice site (*). (C) At 2 dpf, *dnm2* MO-injected embryos exhibit shortened body length, upward curled tails, pericardial and yolk edema, and reduced head size when compared to control morpholino injected embryos. By contrast, embryos injected with *dnm2-like* MO have small muscle compartments, pigmentation defects, and mild tail curvature. (D) Percent of affected embryos at 2 dpf (ctl MO vs. *dnm2* MO p<0.0001, ctl MO vs. *dnm2-like* MO p<0.0001; Fisher’s exact test). The total number of embryos is noted above each bar.

To better clarify the roles of *dnm2* and *dnm2-like*, we used targeted morpholino oligonucleotides to knockdown expression of both genes during early development. Morpholinos were targeted to splice junctions in *dnm2* and *dnm2-like* pre-mRNAs (Figure 3A), and the resulting products were confirmed to be out of frame by sequencing the RT-PCR products (Figure 3B). A standard control morpholino was injected for comparison (Gene-Tools).

Both *dnm2* MO (0.3 mM) and *dnm2-like* MO (0.1 mM) injection resulted in pronounced but non-overlapping developmental phenotypes compared to ctl MO (0.3 mM) injection (Figure 3C). Knockdown of Dnm2 caused a shorted body axis, small eyes, yolk and cardiac edema, shortened somites, and an upward tail curvature. Knockdown of Dnm2-like resulted in a thinned body axis, small eyes, and pigmentation defects. The severity and penetrance of morpholino phenotypes was consistent between injections (control *n* = 601, *dnm2 n* = 601, *dnm2-like n* = 587). At 2 dpf, both morpholino groups had a significant increase in abnormal morphology relative to control morpholino (Figure 3D; p<0.0001, Fisher’s exact test); 93% of *dnm2* morphants and 74% of *dnm2-like* morphants exhibited the described phenotypes, while only 4% of control embryos displayed any developmental abnormalities. To determine knockdown of dynamin-2 expression in *dnm2* and *dnm2-like* morphants, isolated muscle fibers were stained with an antibody against dynamin-2. Cells from both *dnm2* and *dnm2-like* morphants had reduced staining relative to control morphants (data not shown).

**Figure 4:**
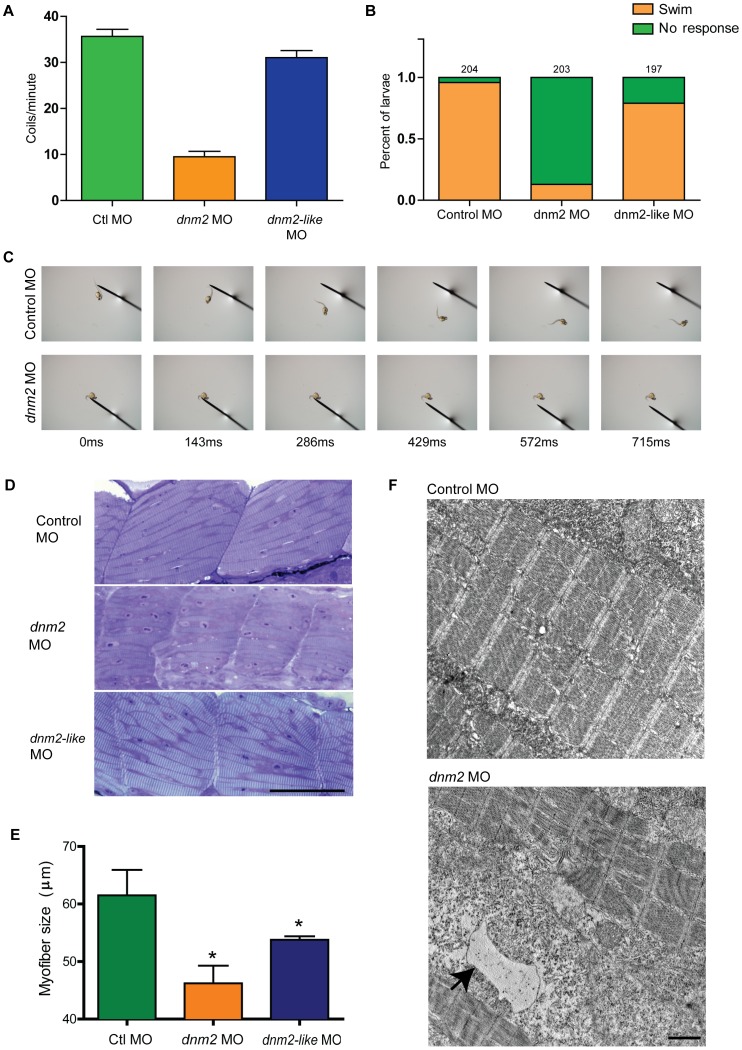
Knockdown of *dnm2* results in motor deficits and abnormal muscle histology. (A) Quantitation of spontaneous embryo coiling at 1 dpf. On average, control morphants coiled 35.7 times in 60 seconds, while *dnm2* morphants coiled only 9.5 times. (B-C) Touch-evoked swimming was measured in 3 dpf morphants. Most control and *dnm2-like* morphants responded to tail taps with a rapid escape response, while *dnm2* morphants exhibited impaired escape responses. (D) Toluidine blue stained semi-thin sections from 3 dpf morphants. Somites from *dnm2* morphants are small with highly disorganized myofibers. Scale bar is equal to 50 µm. (E) Quantification of myofiber length in 3 dpf embryos. Average myofiber size in control embryos equaled 87.8 µm, while *dnm2-like* morphants equaled 76.8 µm and *dnm2* morphants equaled 66.0 µm (*p<0.05 ctl to *dnm2-like*, *p<0.01 ctl to *dnm2,* p = 0.056 *dnm2* to *dnm2-like* morphants; ANOVA ). (F) Representative electron micrographs from larval *dnm2* morphant muscle. Irregular membrane structures were found throughout the muscle (black arrow). Scale bar is equal to 1 µm.

In order to further examine the effect of dynamin-2 depletion on embryonic and larval muscle, we assayed two motor behaviors during development. First, we looked at spontaneous coiling behavior in 1 dpf embryos. Spontaneous coiling is a highly stereotyped behavior detected in zebrafish embryos between approximately 17 and 26 hours post fertilization [19]. Control embryos contracted an average of 35.7±1.5 times per minute and, similarly, *dnm2-like* morphants contracted 31.0±1.6 times per minute (Figure 4A; control *n* = 119, *dnm2-like n* = 107; ns). By contrast, *dnm2* morphants only contracted an average of 9.5±1.2 times per minute (*dnm2 n* = 114; p<0.001, ANOVA). Next, we examined touch-evoked behavior in 3 dpf larvae. At this stage of development, larvae typically respond to a tactile stimulus with a rapid escape response. However, 87.2% of *dnm2* morphants failed to respond to a tail tap stimulus (Figure 4B–C, *n* = 203). Only 4.0% of control morphants and 20.3% of *dnm2-like* morphants did not respond to a tail tap stimulus (control *n* = 204; dnm2-like *n* = 197). Together, the reduced spontaneous coiling and diminished touch-evoked escape behaviors suggests that *dnm2* morphants have a defect in motor function that is not shared by *dnm2-like* morphants.

### Histopatholgical and Ultrastructural Abnormalities in *dnm2* Morphant Muscle

In light of the observed motor defects in *dnm2* morphants, we examined histological and ultrastructural features in muscle from 3 dpf larvae. Semi-thin sections were obtained from the trunks of 3 dpf larvae injected with control, *dnm2*, or *dnm2-like* morpholino (Figure 4D). While sections from *dnm2* morphant muscle revealed striking fiber disorganization, as well as small somites and indistinct striations as compared with control muscle, sections from *dnm2-like* morphant muscle only revealed moderate effects on myofibers. Quantification of myofiber size indicated that fibers from *dnm2* morphants were significantly and substantially smaller than those of control embryos (p<0.009). Myofibers from *dnm2-like* morphants were also significantly smaller than fibers from larvae injected with control morpholino (p<0.05; Figure 4E). The *dnm2* morphant myofibers were, in addition, smaller than those from *dnm2-like* morphants; however, this difference did not reach statistical significance (p = 0.056 for direct comparison of *dnm2* to *dnm2-like*). Similarly, electron microscopy of *dnm2* morphant muscle revealed substantial disorganization with irregular membrane accumulations (Figure 4F; arrow) but only subtle changes in the *dnm2-like* morphants (data not shown). Of note, sarcomeric structures appeared normal in both groups, suggesting that dnm2 is not required for establishing basic myofibril organization.

### Expression of Human DNM2 Rescues *dnm2* and *dnm2-like* Knockdown

**Figure 5:**
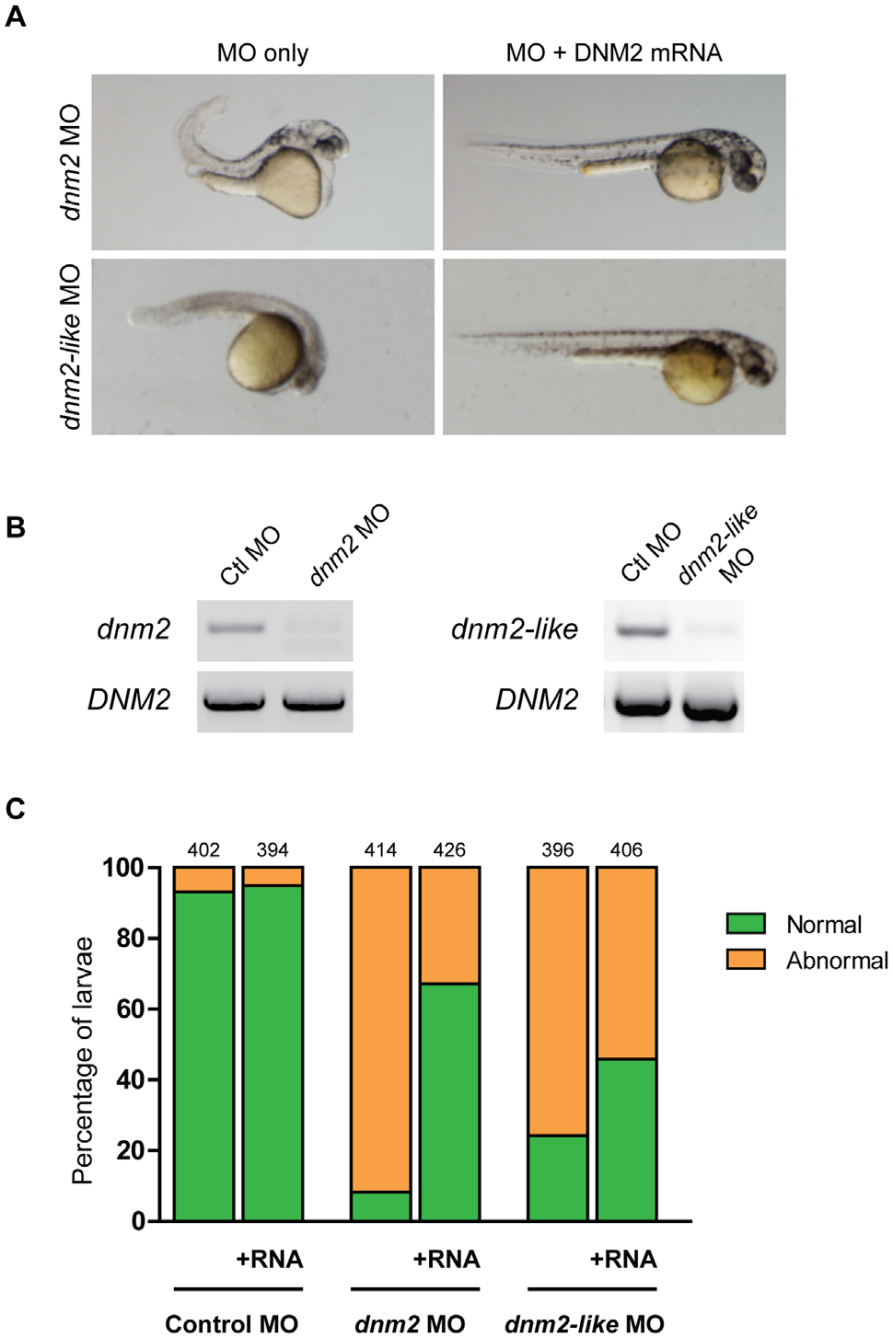
Human DNM2 RNA rescues *dnm2* and *dnm2-like* morphant phenotypes. Rescue of *dnm2* and *dnm2-like* morphants at 2 dpf. (A) Co-injection of human DNM2 RNA can rescue morphological abnormalities in both morphants. (B) RT-PCR of human DNM2 expression in *dnm2* or *dnm2-like* morphants at 3 dpf. (C) The percentage of normal appearing larvae is significantly increased in both *dnm2* and *dnm2-like* rescue conditions, but not in control larvae (*dnm2* p<0.0001, *dnm2-like* p<0.0001, ctl p = 0.30; Fisher’s exact test). The total number of embryos is noted above each bar.

To rescue the *dnm2* and *dnm2-like* morphant phenotypes, embryos were co-injected with human *DNM2* capped mRNA and morpholino at the 1- to 2-cell stage (Figure 5). Expression of DNM2 did not cause any morphological abnormalities in control-injected embryos. At 2 dpf, the percent of normal-appearing embryos was significantly increased in both rescue conditions (control *n* = 796, *dnm2 n* = 840, *dnm2*-*like n* = 802). In *dnm2* morphants, the percent of normal embryos increased from 8.2% to 67.1% (p<0.0001, Fisher’s exact test). In *dnm2-like* morphants, the percent of normal embryos increased from 24.2% to 45.8% (p<0.0001, Fisher’s exact test). Additional rescue experiments with human DNM1 and DNM3 reveal that, although all 3 classical dynamins can rescue the functional defects observed in *dnm2* morphants to a similar extent, only DNM2 had a significant effect on the *dnm2-like* morphant behavior (data not shown). Together, these data support the contention that *dnm2* and *dnm2-like* are functional orthologs of human DNM2.

## Discussion

DNM2 plays an important role in endocytosis and several intracellular membrane trafficking pathways [20]. Given this prominent role in cellular function and the fact that mutations in *DNM2* are associated with two disorders affecting nerve and muscle – Charcot-Marie-Tooth disease and centronuclear myopathy – understanding its specific role in nerve and muscle are critical to enhance our understanding of the role of DNM2 in these tissues in health and disease. *In vitro* and murine models of DNM2-related centronuclear myopathy have begun to shed light on how DNM2 contributes to muscle defects [20], [21]; however, better models are needed to recapitulate disease characteristics and gain more meaningful insight into disease pathogenesis. Zebrafish are becoming an increasingly popular model for the study of muscle disorders; in addition to the many advantages of zebrafish as a model system, zebrafish muscle shares many histological features with mammalian muscle, their neuromuscular system is well-characterized, and various approaches facilitate the development of disease models. As a first step towards developing zebrafish models of DNM2-related neuromuscular disease, this manuscript describes the characterization of two zebrafish dynamin-2 orthologs, as well as the effects of altered gene expression on muscle histology and function.

In this study, we characterize two dynamin-2 genes in the zebrafish genome. The two genes are likely a product of the whole genome duplication that occurred in the ray fin fish lineage prior to the evolution of the teleost [22], [23]. The syntenic organization of both genes supports this conclusion. *dnm2* (zebrafish chromosome 3) shares close syntenic conservation with *DNM2* (human chromosome 19), as it is directly flanked by homologs of the upstream and downstream neighbors of human *DNM2* (*TMED1* and *QTRT1*). While *dnm2-like* (zebrafish chromosome 1) does not share this immediate syntenic block, the human homologs of at least four nearby genes are found within a 0.5 Mb distance of human *DNM2* (*TMED1*, *CDC37*, *OLFM2*, *COL5A3* and *RDH8*). Additionally, both zebrafish genes are found near chromosomal regions that have previously been reported to share homology with human chromosome 19 [24].

At both the gene and protein level, *dnm2* and *dnm2-like* share structural similarity with human *DNM2*. All three genes have a similar intron-exon organization, although *dnm2-like* has much smaller introns. Shrinkage of introns has been reported in several other teleost homologs to human genes [25], [26], [27]. At the protein level, the predicted amino acid sequences of Dnm2 and Dnm2-like share a high percent identity to human DNM2, as well as to each other. When we examined the DNA sequence of other human and zebrafish classical dynamins, phylogenetic analysis grouped *dnm2* and *dnm2-like* with *DNM2* rather than *DNM1* or *DNM3*.

Mammalian DNM2 is ubiquitously expressed in adult tissue [7], [8], [9]. In zebrafish, we found *dnm2* and *dnm2-like* expression in every tissue we examined, which suggests these genes may also be ubiquitously expressed. Both genes were also expressed throughout early development. The early presence of these gene products makes it likely that *dnm2* and *dnm2-like* mRNAs are maternally deposited. This contention is further supported by our observations following knockdown of either *dnm2* or *dnm2-like*. Both morpholino reagents used in this study are splice-targeting morpholinos which only target unprocessed mRNA transcripts; therefore, expression of maternally deposited mRNAs will not be knocked down by the morpholino oligonucleotides. Since we detect *dnm2* and *dnm2-like* mRNA at the one-cell stage, it is likely that both gene products are unaffected by morpholino knockdown during the first few hours of development. In spite of this, we see distinct morphological defects in both *dnm2* and *dnm2-like* morphants by 1 dpf. However, future studies assessing markers of muscle development and function will be required to ascertain the precise impact of morpholino-mediated knockdown in these embryos on muscle development.

Our current findings indicate that both morphological and functional abnormalities are present in zebrafish embryos following *dnm2* and *dnm2-like* knockdown. Morphologically, *dnm2* morphants exhibited a shortened body axis, upward tail curvature, small head size, and edema, while *dnm2-like* morphants displayed only mild tail curvature along with small muscle compartments and pigmentation defects. Further analyses of muscle histology revealed significant effects of both *dnm2* and *dnm2-like* knockdown on myofiber length. The effects on fiber length in *dnm2* morphants were greater than those observed in *dnm2-like* morphants, and *dnm2* morphant embryos also exhibit irregular membrane structures upon EM. Similar histopathological changes in muscle have been previously described [28] and further support is provided by Durieux et al, who demonstrate decreased muscle size in transgenic mice heterozygous for mutant R465W-*Dnm2,* and Laporte et al, who describe histopathological features including centralized nuclei and fiber atrophy with adenoviral overexpression of R465W-DNM2 in adult mouse muscle [29], [30]. Interestingly, expression of R465W-DNM2 in this model was initiated in adult muscle, demonstrating that DNM2 plays an important role in muscle maintenance after myogenesis. Despite the occurrence of morphological effects in both *dnm2* and *dnm2-like* morphants, however, behavioral characterization reveals a more varied effect on muscle function. *dnm2* morphants exhibit a striking deficit in both spontaneous and touch-evoked escape behavior, while *dnm2-like* morphants exhibit relatively mild phenotypes in this regard. Similar effects of mutant *Dnm2* have been reported in heterozygous R465W-*Dnm2* mice, which exhibit reductions in muscle force by 3 weeks of age [30]. Together, the current zebrafish data, along with insight gained from previously reported mutant *Dnm2* mouse models, confirm a role for DNM2 in muscle structure and function.

In order to further support the hypothesis that *dnm2* and *dnm2-like* share functional homology with human DNM2, we then examined the ability of human *DNM2* to rescue the phenotype of both *dnm2* and *dnm2-like* morphants. Human *DNM2* expression can partially rescue the phenotypes resulting from knockdown of *dnm2* and *dnm2-like*. This reduction in the fraction of abnormal embryos was greater for *DNM2* rescue of the *dnm2* morphants, although significant improvements were also seen for *dnm2-like* morphants co-injected with *DNM2* RNA. Interestingly, additional rescue experiments in the zebrafish with human dynamins (data not shown) have also demonstrated that touch-evoked escape responses in the *dnm2* morphants can be rescued by expression of *DNM1, DNM2* or *DNM3,* while only *DNM2* is able to convincingly rescue swimming behavior in *dnm2-like* morphants. This may suggest that *dnm2-like* is the more closely related *DNM2* ortholog in zebrafish. However, previous studies have shown that the classical dynamins can co-oligomerize [31], [32], and *in vitro* and knockout mouse studies both show that DNM1 and DNM3 can compensate for DNM2 loss [33]. Therefore, we are unable to conclusively distinguish *dnm2* or *dnm2-like* as more closely resembling human *DNM2*, and maintain that both are likely orthologs of the human gene.

Together, our data support a functional connection between the *dnm2* and *dnm2-like* orthologs in zebrafish; however, despite similar expression patterns and effects of *dnm2* and *dnm2-like* knockdown on zebrafish muscle histology, the varying severity of these phenotypes along with the differential effects on functional assessments indicates that they play both overlapping and distinct roles in zebrafish muscle. On one hand, the observed functional differences could be due to differences in knockdown efficiency between the *dnm2* and *dnm2-like* morphants. Alternatively, gene-specific functional differences could exist. Future gene-specific targeting and mutant DNM2 studies addressing the detailed mechanisms responsible for the observed histological and functional deficits in morphant zebrafish are warranted to comprehend the exact role these proteins are playing in muscle development and function. For example, activity-deficient DNM2 mutants could be employed to assess the contribution of enzymatic activity on endocytosis and muscle structure and function. Electrophysiological studies may also provide insight into the correlation of the observed morphological defects with functional outcomes. Finally, studies assessing potential disease-causing mechanisms may be required to understand the role of DNM2 in disease. Endocytosis and autophagy defects, altered oligomerization, abnormalities in muscle membrane structure development and maintenance, and effects at the neuromuscular junction are all important mechanisms [29], [30], [34], [35] to consider and investigate to determine how DNM2 contributes to neuromuscular disorders.

Taken together, our findings show that *dnm2* and *dnm2-like* are highly conserved orthologs to human *DNM2* are independently required for normal embryonic development in the zebrafish. It will be important to further examine these two genes in order to understand their specific cellular function in the zebrafish. The zebrafish provides an excellent system for examining aspects of membrane trafficking *in vivo*, and understanding the zebrafish dynamin-2 homologs will allow a more precise analysis of these pathways.

## Supporting Information

Figure S1 **Zebrafish** ***dnm2*** **whole mount** ***in situ*** **hybridization.** (A) Whole mount *in situ* of 1 dpf embryos reveals ubiquitous expression of dnm2. (B) Sense probe to dnm2 was used as a background control.
(TIF)

## References

[1] Pucadyil TJ, Schmid SL (2009) Conserved functions of membrane active GTPases in coated vesicle formation. Science 325: 1217–1220. 19729648 10.1126/science.1171004 PMC2864031

[2] Faelber K, Posor Y, Gao S, Held M, Roske Y, et al (2011) Crystal structure of nucleotide-free dynamin. Nature 477: 556–560. 21927000 10.1038/nature10369

[3] Ford MG, Jenni S, Nunnari J (2011) The crystal structure of dynamin. Nature 477: 561–566. 21927001 10.1038/nature10441 PMC4075756

[4] Gold ES, Underhill DM, Morrissette NS, Guo J, McNiven MA, et al (1999) Dynamin 2 is required for phagocytosis in macrophages. J Exp Med 190: 1849–1856. 10601359 10.1084/jem.190.12.1849 PMC2195719

[5] Henley JR, Krueger EW, Oswald BJ, McNiven MA (1998) Dynamin-mediated internalization of caveolae. J Cell Biol 141: 85–99. 9531550 10.1083/jcb.141.1.85 PMC2132718

[6] Jones SM, Howell KE, Henley JR, Cao H, McNiven MA (1998) Role of dynamin in the formation of transport vesicles from the trans-Golgi network. Science 279: 573–577. 9438853 10.1126/science.279.5350.573

[7] Sontag JM, Fykse EM, Ushkaryov Y, Liu JP, Robinson PJ, et al (1994) Differential expression and regulation of multiple dynamins. J Biol Chem 269: 4547–4554. 8308025

[8] Diatloff-Zito C, Gordon AJ, Duchaud E, Merlin G (1995) Isolation of an ubiquitously expressed cDNA encoding human dynamin II, a member of the large GTP-binding protein family. Gene 163: 301–306. 7590285 10.1016/0378-1119(95)00275-b

[9] Cook TA, Urrutia R, McNiven MA (1994) Identification of dynamin 2, an isoform ubiquitously expressed in rat tissues. Proc Natl Acad Sci U S A 91: 644–648. 8290576 10.1073/pnas.91.2.644 PMC43005

[10] Ferguson SM, Raimondi A, Paradise S, Shen H, Mesaki K, et al (2009) Coordinated actions of actin and BAR proteins upstream of dynamin at endocytic clathrin-coated pits. Dev Cell 17: 811–822. 20059951 10.1016/j.devcel.2009.11.005 PMC2861561

[11] Bitoun M, Maugenre S, Jeannet PY, Lacene E, Ferrer X, et al (2005) Mutations in dynamin 2 cause dominant centronuclear myopathy. Nat Genet 37: 1207–1209. 16227997 10.1038/ng1657

[12] Zuchner S, Noureddine M, Kennerson M, Verhoeven K, Claeys K, et al (2005) Mutations in the pleckstrin homology domain of dynamin 2 cause dominant intermediate Charcot-Marie-Tooth disease. Nat Genet 37: 289–294. 15731758 10.1038/ng1514

[13] Ahn HJ, Park Y, Kim S, Park HC, Seo SK, et al (2010) The expression profile and function of Satb2 in zebrafish embryonic development. Mol Cells 30: 377–382. 20814748 10.1007/s10059-010-0128-6

[14] Kida YS, Sato T, Miyasaka KY, Suto A, Ogura T (2007) Daam1 regulates the endocytosis of EphB during the convergent extension of the zebrafish notochord. Proc Natl Acad Sci U S A 104: 6708–6713. 17412835 10.1073/pnas.0608946104 PMC1871850

[15] Feng B, Schwarz H, Jesuthasan S (2002) Furrow-specific endocytosis during cytokinesis of zebrafish blastomeres. Exp Cell Res 279: 14–20. 12213209 10.1006/excr.2002.5579

[16] Tamura K, Peterson D, Peterson N, Stecher G, Nei M, et al (2011) MEGA5: molecular evolutionary genetics analysis using maximum likelihood, evolutionary distance, and maximum parsimony methods. Mol Biol Evol 28: 2731–2739. 21546353 10.1093/molbev/msr121 PMC3203626

[17] Kwan KM, Fujimoto E, Grabher C, Mangum BD, Hardy ME, et al (2007) The Tol2kit: a multisite gateway-based construction kit for Tol2 transposon transgenesis constructs. Dev Dyn 236: 3088–3099. 17937395 10.1002/dvdy.21343

[18] Dowling JJ, Vreede AP, Low SE, Gibbs EM, Kuwada JY, et al (2009) Loss of myotubularin function results in T-tubule disorganization in zebrafish and human myotubular myopathy. PLoS Genet 5: e1000372. 19197364 10.1371/journal.pgen.1000372 PMC2631153

[19] Saint-Amant L, Sprague SM, Hirata H, Li Q, Cui WW, et al (2008) The zebrafish ennui behavioral mutation disrupts acetylcholine receptor localization and motor axon stability. Dev Neurobiol 68: 45–61. 17918238 10.1002/dneu.20569

[20] Durieux AC, Prudhon B, Guicheney P, Bitoun M (2010) Dynamin 2 and human diseases. J Mol Med (Berl) 88: 339–350. 20127478 10.1007/s00109-009-0587-4

[21] Cowling BS, Toussaint A, Muller J, Laporte J (2012) Defective membrane remodeling in neuromuscular diseases: insights from animal models. PLoS Genet 8: e1002595. 22496665 10.1371/journal.pgen.1002595 PMC3320571

[22] Taylor JS, Braasch I, Frickey T, Meyer A, Van de Peer Y (2003) Genome duplication, a trait shared by 22000 species of ray-finned fish. Genome Res 13: 382–390. 12618368 10.1101/gr.640303 PMC430266

[23] Postlethwait J, Amores A, Cresko W, Singer A, Yan YL (2004) Subfunction partitioning, the teleost radiation and the annotation of the human genome. Trends Genet 20: 481–490. 15363902 10.1016/j.tig.2004.08.001

[24] Naruse K, Tanaka M, Mita K, Shima A, Postlethwait J, et al (2004) A medaka gene map: the trace of ancestral vertebrate proto-chromosomes revealed by comparative gene mapping. Genome Res 14: 820–828. 15078856 10.1101/gr.2004004 PMC479108

[25] Long QM, Meng AM, Wang H, Jessen JR, Farrell MJ, et al (1997) GATA-1 expression pattern can be recapitulated in living transgenic zebrafish using GFP reporter gene. Development 124: 4105–4111. 9374406 10.1242/dev.124.20.4105

[26] Daniels GD, Secombes CJ (1999) Genomic organisation of rainbow trout, Oncorhynchus mykiss TGF-beta. Dev Comp Immunol 23: 139–147. 10227481 10.1016/s0145-305x(98)00051-2

[27] Pugacheva EM, Kwon YW, Hukriede NA, Pack S, Flanagan PT, et al (2006) Cloning and characterization of zebrafish CTCF: Developmental expression patterns, regulation of the promoter region, and evolutionary aspects of gene organization. Gene 375: 26–36. 16647825 10.1016/j.gene.2006.01.036

[28] Nicot AS, Toussaint A, Tosch V, Kretz C, Wallgren-Pettersson C, et al (2007) Mutations in amphiphysin 2 (BIN1) disrupt interaction with dynamin 2 and cause autosomal recessive centronuclear myopathy. Nat Genet 39: 1134–1139. 17676042 10.1038/ng2086

[29] Cowling BS, Toussaint A, Amoasii L, Koebel P, Ferry A, et al (2011) Increased expression of wild-type or a centronuclear myopathy mutant of dynamin 2 in skeletal muscle of adult mice leads to structural defects and muscle weakness. Am J Pathol 178: 2224–2235. 21514436 10.1016/j.ajpath.2011.01.054 PMC3081151

[30] Durieux AC, Vignaud A, Prudhon B, Viou MT, Beuvin M, et al (2010) A centronuclear myopathy-dynamin 2 mutation impairs skeletal muscle structure and function in mice. Hum Mol Genet 19: 4820–4836. 20858595 10.1093/hmg/ddq413

[31] Lu J, Helton TD, Blanpied TA, Racz B, Newpher TM, et al (2007) Postsynaptic positioning of endocytic zones and AMPA receptor cycling by physical coupling of dynamin-3 to Homer. Neuron 55: 874–889. 17880892 10.1016/j.neuron.2007.06.041 PMC2597538

[32] Solomaha E, Palfrey HC (2005) Conformational changes in dynamin on GTP binding and oligomerization reported by intrinsic and extrinsic fluorescence. Biochem J 391: 601–611. 15954862 10.1042/BJ20050707 PMC1276961

[33] Ferguson SM, De Camilli P (2012) Dynamin, a membrane-remodelling GTPase. Nat Rev Mol Cell Biol 13: 75–88. 22233676 10.1038/nrm3266 PMC3519936

[34] Durieux AC, Vassilopoulos S, Laine J, Fraysse B, Brinas L, et al (2012) A centronuclear myopathy–dynamin 2 mutation impairs autophagy in mice. Traffic 13: 869–879. 22369075 10.1111/j.1600-0854.2012.01348.x

[35] Bitoun M, Durieux AC, Prudhon B, Bevilacqua JA, Herledan A, et al (2009) Dynamin 2 mutations associated with human diseases impair clathrin-mediated receptor endocytosis. Hum Mutat 30: 1419–1427. 19623537 10.1002/humu.21086

