# Supplementary figures and images for: Two Dynamin-2 Genes Are Required for Normal Zebrafish Development
Source: PLoS One. 2013 Feb 13;8(2):e55888. doi: 10.1371/journal.pone.0055888 (PMC3572173; doi:10.1371/journal.pone.0055888)

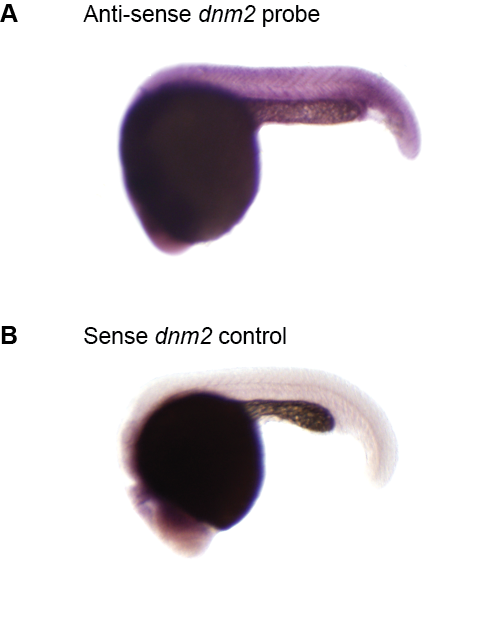

Supplement: Figure S1 — Zebrafish dnm2 whole mount in situ hybridization. (A) Whole mount in situ of 1 dpf embryos reveals ubiquitous expression of dnm2. (B) Sense probe to dnm2 was used as a background control. (TIF) [file pone.0055888.s001.tif]
